# Hospital Festivals, Meetings, &c.

**Published:** 1894-03-31

**Authors:** 


### HOSPITAL FESTIVALS, MEETINGS, ETC.

### ROYAL LONDON OPHTHALMIC HOSPITAL.

The annual general meeting was held at this hospital on March 20th. The chair was taken by Mr. Charles Gordon, and among those present were the Rev. R. Whittington, Mr. Hawes, Mr. Watts, Mr. H. Heeps, Mr. E. Hogg, and Mr. Shea Smith. The statement of accounts accompanying the annual report showed a considerable excess of income over expenditure, due chiefly to the large sum received from legacies, but the sum of £30,000 is required in order to build a new hospital, the site of which has not yet been determined on. The total number of in-patients admitted during the year was 1,972, of out-patients 24,955, with a total of 116,775 attendances. The report having been adopted, the thanks of the meeting was voted to the President of the hospital (Sir John Lubbock), to the Treasurer (Mr. John Deacon), the Chairman, as well as to the Secretary of the hospital (Mr. Robert Newstead).

### UNIVERSITY COLLEGE HOSPITAL.

The annual meeting of governors and subscribers was held at this hospital on March 21st, the chair being taken by Mr. Henry Lucas. The annual report presented to the meeting was not of a very cheering nature. On account of the closing of the Middlesex Hospital for two months, and the partial closing of other hospitals, there has been a large increase in the number both of in and out-patients received, which has not been met by a corresponding increase of income. In fact, as the institution is already very deeply in debt, the committee see no other way of getting out of their difficulties, if more help be not forthcoming, than by reducing the number of beds. The Chairman, in moving the adoption of the report, spoke strongly of the loss which such a partial closing would be to the neighbourhood, as already many patients have to be turned away for want of room. He also regretted the slow progress made by the Rebuilding Fund, which has not yet reached £30,000, the sum fixed upon to be received before rebuilding can be commenced. The report further gave the number of in-patients in 1893 as 3,228, and of out-patients as 49,486, in both cases a considerable increase over those of the year before.

